# Hydrodissection for the Treatment of Vascular Thoracic Outlet Syndrome

**DOI:** 10.7759/cureus.29229

**Published:** 2022-09-16

**Authors:** John M Ver Hoef, Daniel Clearfield

**Affiliations:** 1 Texas College of Osteopathic Medicine, University of North Texas Health Science Center, Fort Worth, USA; 2 Sports Medicine, Motion is Medicine, Fort Worth, USA

**Keywords:** thoracic outlet, intermittent claudication, hydrodissection, conservative medical management, arterial thoracic outlet syndrome

## Abstract

The following case explores the effectiveness of a new treatment modality for vascular thoracic outlet syndrome (vTOS). Few conservative treatments exist to alleviate symptoms of vTOS. In this case, a 25-year-old male was diagnosed with vTOS four months prior to presentation. A combination of poor posture, inactivity, and protruding screws from a prior clavicle fracture repair were compromising the subclavicular vasculature. Symptoms of claudication and a cold right arm/hand led the patient to seek medical treatment. Post failed physiotherapy and pharmacotherapy, the vascular surgeon advised for surgery. He was hesitant to undergo major surgical intervention; therefore, after exploring possible remedies, hydrodissection was chosen for its potential merit in this case. After hydrodissection was performed to decompress the subclavicular neurovascular bundle, the patient reported immediate alleviation of his symptoms. Post two-week and three-month follow-up, there was complete resolution of symptoms with no recurrence. Although there is a lack of literature supporting the use of hydrodissection to treat vTOS, this was a specific case in which hydrodissection demonstrated to be an effective treatment modality. The specific utilization of hydrodissection should be further studied to increase the literature base and increase awareness of its potential effectiveness for this and similar conditions.

## Introduction

Thoracic outlet syndrome (TOS) has many different presentations. TOS results from the compression of the neurovascular bundle to the arm and can present with neuropathic symptoms, ischemic symptoms, or both [1]. In cases of arterial TOS (aTOS), the compression of the subclavian artery can originate between the anterior and the medial scalene muscles, between the clavicle and the first rib, beneath the pectoralis minor, or from a cervical rib [2]. In recent years, sufficient technology exists to properly pinpoint and diagnose certain types of TOS.

Arterial TOS represents only 1% of all TOS diagnoses. With such little data, exact presentations vary, and treatments for the various causes of aTOS are determined on a case-by-case basis. However, commonalities across diagnoses are the symptoms during presentation. Patients often report a cold extremity, pallor, and ischemic pain during activity (termed claudication). Often, aTOS is attributable to structural anomalies, but it can also include peripheral artery disease or a thrombogenic problem [1]. Diagnosis relies largely on imaging such as ultrasound, CT/MRI, and angiography/arteriography.

One specific cause of aTOS can be due to previous clavicular fractures, which lead to the development of fracture calluses [3] or, in the case of repair, plates and screws in close proximity to the neurovasculature. A few case reports have discussed iatrogenic TOS, but few cover aTOS in the setting of previous clavicle fracture repair. This case report will discuss the diagnosis and management of aTOS resulting from inflammation and screws with callouses protruding into the subclavicular neurovascular bundle.

Treatment for aTOS resulting from impingement usually relies on surgical decompression or the removal of the offending agent. In this case, surgery was not an option due to patient preferences. The alternative treatment of hydrodissection has been utilized in a few cases of neurogenic TOS [4]. Hydrodissection is a minimally invasive method of creating space around impinged structures by injecting fluid into the surrounding area to provide relief. The procedure is commonly used for creating planes between tissues or lysing adhesions. Although little to no literature exists on the specific use of hydrodissection in the case of aTOS, the idea of using a solution to decompress the neurovascular bundle has merit. This study will focus on the results of using hydrodissection to relieve aTOS in a patient with subclavicular compression. This information will be valuable in providing a more conservative approach to the treatment of aTOS.

## Case presentation

A 25-year-old male presents to the sports medicine clinic in April 2021 with a four-month history of intermittent loss of pulse in the right arm. He first noticed his right hand becoming cold, with associated pallor and claudication. Later, he discovered the pulse in his radial artery disappearing during provocative positions such as external rotation with the abduction of the arm or protraction of the shoulders. At the age of 15, the patient suffered a right collar bone fracture repaired with a plate and six screws. He remained active and had no symptoms of TOS until four months before the presentation. The patient believed that his problems were due to being inactivity and having poor posturing for so long due to COVID confining him indoors. The patient has tried immobilization and nonsteroidal anti-inflammatory drugs (NSAIDs) and reported that neither of these alleviated his symptoms. Since the onset of the symptoms, the patient reports they have increasing frequency and severity, which have led him to seek medical evaluation.

During his first medical visit with a vascular surgeon, the patient was assessed and underwent an electromyography (EMG) study, Doppler ultrasound, and blood pressure testing in both arms. During the physical examination, the loss of pulse was confirmed with provocative testing such as Adson’s test, Roo’s test, and Wright’s hyperabduction test. The EMG study confirmed the slowing of conduction in the brachial plexus in the shoulder region. The Doppler ultrasound was non-contributory. The blood pressure recorded on the right arm was the same as the left in normal posture positioning, but during positions such as protraction of the shoulders, abduction and external rotation of the arm, or shoulder extension, both systolic and diastolic pressures markedly decreased. The combination of these findings confirmed a diagnosis of TOS with both arterial and neurological components. The patient’s focus was on the arterial symptoms, which was the target for treatment. The treatment recommended by the vascular surgeon was the removal of the anterior and middle scalene muscles, the first rib, and the pectoralis minor, a procedure that did not align with the patient’s goal of not undergoing any invasive surgery and was likely unnecessary and overly invasive for the patient’s problem. After declining the surgery, the patient sought more conservative treatment and was enrolled in physical therapy (PT) for the shoulder. The PT regimen included one-hour sessions three times per week for four weeks. The sessions included strength and range of motion training with supplemental therapeutic massage. After four weeks of PT, the patient was still bothered by the aTOS symptoms and sought a second opinion.

Upon presentation to the sports medicine clinic, the patient reported no improvement in claudication or coldness of the extremity. The patient had no significant past medical history. On clinical examination, the upper extremities demonstrated normal tone, strength, motor function, and reflexes. The right hand was notably colder to palpation than the left, and the right radial pulse ranged from 0/4 to 1/4 in provocative positions but 2/4 when in a neutral position. Ultrasound was utilized to attempt to pinpoint the area of stenosis. With the probe underneath the right clavicle, screws were seen protruding 2-4 mm out of the inferior surface. Local edema was noted around the subclavian artery below the clavicle. Areas of arterial constriction were noted below the clavicle and pectoralis minor. Given the lack of change in symptoms with PT, immobilization, or pharmacotherapy, hydrodissection was discussed and ultimately performed on the patient, with the goal of separating the artery from the surrounding structures to reduce irritation.

For the treatment, three areas were hydrodissected. Using a 22-gauge, 2.75-inch needle, a 20 cc solution that comprised of 9:1 D5W:1% lidocaine was injected into the area surrounding the axillary artery deep to the pectoralis minor via the anterior in-plane approach in a lateral to medial direction. Using a 22-gauge, 1.5-inch needle, another 10 cc of the aforementioned solution was injected into the area surrounding the subclavian artery deep to the subclavius muscle via the anterior in-plane approach in a lateral to medial direction. Using a 22-gauge, 1.5-inch needle, a 10 cc solution of nine parts D5W and one part of 1% lidocaine was injected into the area surrounding the brachial plexus over the clavicle in the area of the surgical screws via the supero-anterior in-plane approach from lateral to medial. All injections were performed under ultrasound guidance with image capture showing the location and spread of the solution. No adverse events were reported.

Minutes after the procedure, the patient reported relief of ischemic symptoms, and the coldness of the right hand was alleviated. A few days after the procedure, the patient resumed his original PT program. Two weeks later, the patient returned for follow-up and reported complete alleviation of the aTOS. The patient reported no further instances of claudication or coldness in the right arm. At three months, the patient maintained complete resolution of the aTOS symptoms.

## Discussion

**Figure 1:**
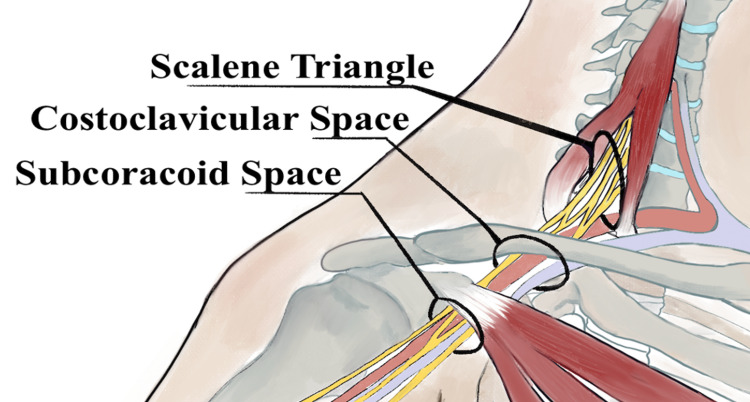
Common areas for TOS to occur TOS: thoracic outlet syndrome The original art was provided by Safa Khawaja.

There are multiple possible compression points that lead to TOS. The first and most common is between an anomalous/cervical rib and the first thoracic rib. Other causes include the point where the subclavian artery and brachial plexus pass through the anterior and medial scalene muscles (scalene triangle), the space between the clavicle and the first rib (costoclavicular space), and the space between the pectoralis minor and ribs 2-4 (subcoracoid space) (Figure 1) [5].

**Figure 2:**
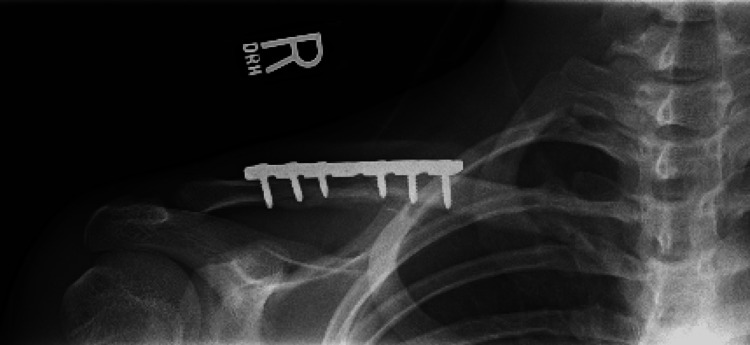
X-ray of the patient’s right clavicle (September 2010) showing the clavicle fixation screws in the patient’s right shoulder

In this case, the patient was experiencing stenosis of the subclavian/axillary artery in the costoclavicular and subcoracoid spaces. Given the complexity and proximity of the potential causes of TOS, the diagnosis may have uncommon and multiple etiologies contributing to the patient’s diagnosis. This is referred to as double crush syndrome [6]. Diagnosis in the sports medicine clinic focused on using ultrasound to find the specific location of arterial compression (Figure 2). In a neutral position, no obvious compression was noted. Screws from the clavicle repair hardware and the respective callouses protruded into the costoclavicular space, narrowing the overall space for the neurovascular bundle to occupy. However, the hardware did not cause obvious compression in a neutral position. When shoulder abduction was introduced, the subclavicular and axillary arteries were narrowed in the costoclavicular and subcoracoid spaces, respectively. This compression was reproducible by the other provocative tests used (Adson’s, Roo’s, and Wright’s hyperabduction tests). In this patient, all three tests elicited a positive result.

To perform Adson’s test, the practitioner palpates the patient’s radial pulse and then abducts the arm while extending the shoulder. The patient’s head is turned toward the ipsilateral arm being tested. Any changes in the radial pulse are detected by the administrator, and any symptoms of compressed nerves are reported by the patient. Symptoms of neurovascular compromise indicate a positive test result.

The Roo’s test involves the patient assuming a “field goal” position by externally rotating and abducting both shoulders to 90 degrees and flexing the arms to 90 degrees. The patient then proceeds to squeeze and release their grip until symptoms of claudication or nerve compression are reported. Symptoms of neurovascular compromise indicate a positive test result.

Lastly, the hyperabduction test is performed by palpating the radial pulse and passively abducting the patient’s arm from neutral to 180 degrees while keeping the elbow in extension. Neurogenic symptoms are reported by the patient, and changes in radial pulse are noted by the administrator. Symptoms of neurovascular compromise indicate a positive test result.

A unique factor in this case is that usually when TOS results after clavicle surgery, symptoms are noticed immediately due to direct impingement of the sensitive structures. This patient had clavicle surgery in 2010 and was only recently noticing symptoms, insisting that the progression of his condition was most likely due to other factors. The patient claimed that it was due to his immobility during the COVID pandemic, which forced him to stay home and adopt a more sedentary lifestyle. Given that there were no acute changes in his anatomy surrounding the onset of symptoms, an invasive surgical procedure seemed to be an extreme solution. After the failure of the first month of PT, progression to minimally invasive treatment with hydrodissection would have potential value in the treatment course.

**Figure 3:**
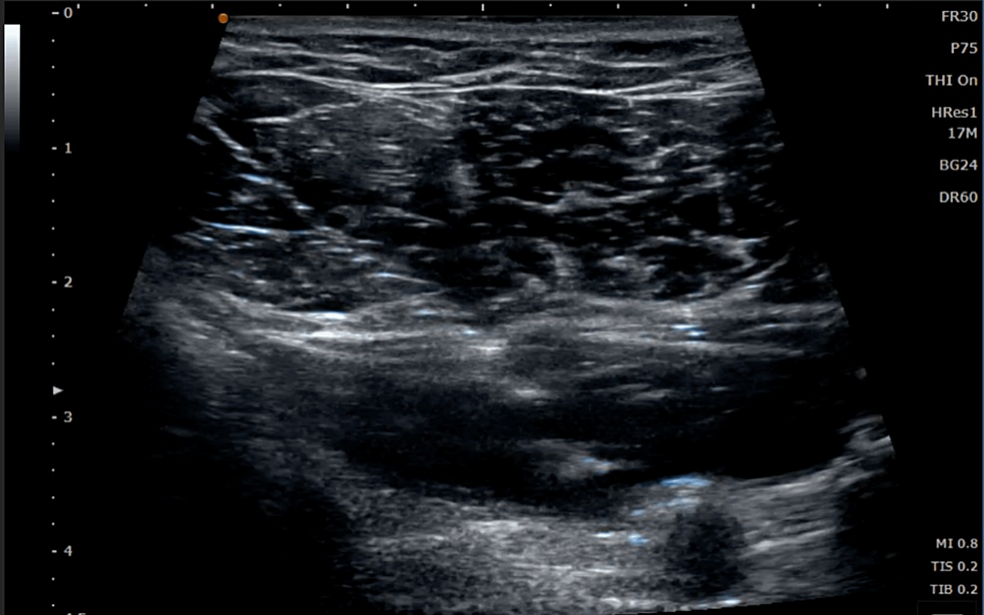
Ultrasound image showing hydrodissection of the patient’s brachial artery in subcoracoid space beneath the pectoralis muscles

**Figure 4:**
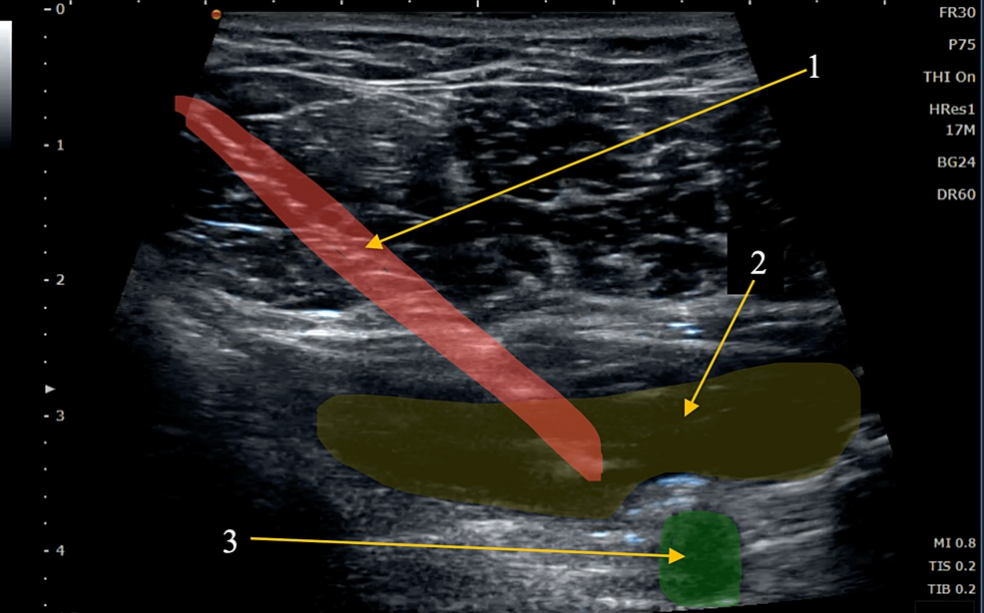
Hydrodissection of the patient’s brachial artery beneath the pectoralis muscles Arrow 1: needle (red), arrow 2: hydrodissection space from injected fluid (yellow), arrow 3: brachial artery (green)

Hydrodissection in this case was used to decompress the subclavian artery as it courses through the shoulder (Figures 3, 4). By creating a tunnel of space between the artery and the surrounding structures, the potential for compression or contact with irritating structures was reduced. Immediately after the procedure, the patient reported alleviation of his symptoms, and with continued postural correction and self-directed exercise, the effects have been lasting.

Literature and case reports regarding hydrodissection for aTOS are lacking. However, this report indicates that the hydrodissection technique could have clinical value in a conservative course of treatment for various types of TOS.

## Conclusions

This study reviews a case of the therapeutic benefit when utilizing ultrasound-guided hydrodissection for the treatment of aTOS. By using this outpatient procedure with local anesthesia, lasting relief of the patient’s symptoms was achieved using a minimally invasive technique. Given the versatility of hydrodissection for other conditions such as lysing adhesions and nerve entrapment, its value should be exercised by more practitioners to help increase the amount of literature and further solidify its usefulness. In the view of this case, hydrodissection should be a consideration in relief of TOS when other conservative treatments have failed.
